# Simultaneous Determination of Sitagliptin Phosphate Monohydrate and Metformin Hydrochloride in Tablets by a Validated UPLC Method

**DOI:** 10.3797/scipharm.1110-13

**Published:** 2011-12-12

**Authors:** Chellu S. N. Malleswararao, Mulukutla V. Suryanarayana, Khagga Mukkanti

**Affiliations:** 1 Analytical Research and Development, Integrated Product Development, Dr. Reddy’s Laboratories Ltd., Bachupally, Hyderabad-500 072, India; 2 Center for Pharmaceutical Sciences, IST, J. N. T. University, Kukatpally, Hyderabad-500 072, India

**Keywords:** Sitagliptin phosphate monohydrate, Metformin hydrochloride, UPLC, Simultaneous, Stability-indicating method, Validation, Chromatography

## Abstract

A novel approach was used to develop and validate a rapid, specific, accurate and precise reverse phase ultra performance liquid chromatographic (UPLC) method for the simultaneous determination of Sitagliptin phosphate monohydrate and Metformin hydrochloride in pharmaceutical dosage forms. The chromatographic separation was achieved on Aquity UPLC BEH C8 100 × 2.1 mm, 1.7 μm, column using a buffer consisting of 10 mM potassium dihydrogen phosphate and 2 mM hexane-1-sulfonic acid sodium salt (pH adjusted to 5.50 with diluted phosphoric acid) and acetonitrile as organic solvent in a gradient program. The flow rate was 0.2 mL min^−1^ and the detection wavelength was 210 nm. The limit of detection (LOD) for Sitagliptin phosphate monohydrate and Metformin hydrochloride was 0.2 and 0.06 μg mL^−1^, respectively. The limit of quantification (LOQ) for Sitagliptin phosphate monohydrate and Metformin hydrochloride was 0.7 and 0.2 μg mL^−1^, respectively. This method was validated with respect to linearity, accuracy, precision, specificity and robustness. The method was also found to be stability-indicating.

## Introduction

Sitagliptin phosphate monohydrate (SP) chemically, (3*R*)-3-amino-1-[3-(trifluoromethyl)-5,6-dihydro[1,2,4]triazolo[4,3-*a*]pyrazin-7(8*H*)-yl]-4-(2,4,5-trifluorophenyl)butan-1-one phosphate hydrate [1] is an oral anti-diabetic, which is available in 25 mg, 50 mg and 100 mg tablets for oral administration. SP is used for the improvement of glycemic control in patients with type II diabetes mellitus as monotherapy or combination therapy with metformin or a peroxisome proliferatoractivated receptor gamma (PPAR) agonist (e.g., thiazolidinediones) when the single agent does not provide adequate glycemic control.

Metformin hydrochloride (MH) chemically, 3-(diaminomethylidene)-1,1-dimethylguanidine hydrochloride [2] is an antidiabetic agent [3]. It is the drug of choice for the treatment of type II diabetes, particularly in overweight and obese people and individuals with normal kidney function. It works by lowering blood sugar and helping the body use insulin more efficiently. It is available in 500 mg, 850 mg and 1000 mg tablets (immediate release) and in 500 mg and 750 mg (slow release) for oral administration. Merck and Co. market SP in combination with MH in a single dosage form as Junumet™ [4]. In combination these are available in 50/500 mg and 50/1000 mg of SP and MH, respectively. The chemical structures of MH, Metformin impurity-1, Metformin impurity-2, SP and Sitagliptin impurity are presented in Tab. 1.

Ultra performance liquid chromatography (UPLC) is an innovative product that brought revolution in high performance liquid chromatography by outperforming conventional HPLC. UPLC provides the speed by using novel low micron particles that decreases chromatographic run times and also double peak capacity or resolution. The current method can be considered a green method because it uses eco-friendly, innovative UPLC technology that reduces the consumption of organic solvent, resulting in less waste. The reduction of the flow rate drastically reduces the mobile phase consumption, thus having obvious economic consequences. With significant improvements in resolution, sensitivity and speed can be achieved for chromatographic separations by minimizing the band spreading contributions of both the instrument and the column. UPLC system will eliminate significant time and cost per sample from analytical process while improving the quality of results, and the system allows chromatographers to work at higher efficiencies with a much wider range of linear velocities, flow rates and back pressures. UPLC photodiode array (PDA) detector detects and quantifies lower concentrations of sample analyte, trace impurities with maximum sensitivity and compares spectra across wavelengths and broad concentration ranges. It is easy to identify components that are difficult to detect by conventional HPLC-based methods.

The literature reveals that some methods have been reported for metformin. Few UV spectrophotometric methods [5], HPLC [6–8] and ion-pair HPLC [9] method have been reported for the estimation of MH. SP is not yet official in any of the pharmacopoeia but MH is official in IP [10], BP [11] and USPNF [12]. Literature survey reveals that only LC-MS [13–15] methods were reported for the determination of SP in plasma and urine of humans, rats and dogs Additionally, some reviewed literature describes the spectroflourometric and spectrophotometric methods for the determination of SP in pharmaceutical dosage forms [16]. Also, in the reviewed literature UPLC HPLC method is not reported for the simultaneous estimation of the SP and MH in combined pharmaceutical dosage form. A method for determination of SP either alone or simultaneous with MH in the presence of SP degradation impurity by HPLC [17] is available, but the current method focuses not only on SP related impurities but also the impurities of MH. Because this method consumes less organic solvent, it can be considered a green method. Therefore, it was thought worthwhile to develop a simple, precise, accurate reverse phase ultra performance liquid chromatographic method for the simultaneous estimation of SP and MH in combined tablet dosage form.

## Experimental

### Instrumentation and Chromatographic Conditions

The UPLC system, used for method development, forced degradation studies and method validation was Waters Acquity UPLC™ system equipped with the binary solvent manager, sample manager, column heater module and photodiode array detector (Waters Corporation, Milford, USA). Aquity UPLC BEH C8 (100 × 2.1 mm, 1.7 μm) was used as stationary phase. The mobile phase composition used was the buffer 10mM potassium dihydrogen phosphate and 2 mM hexane-1-sulfonic acid sodium salt (pH adjusted to 5.50 with diluted phosphoric acid) and acetonitrile with gradient program [Time(min)/% acetonitrile): 0/8, 2/8, 4/45, 6/45, 8/8, 10/8]. Prior to use, the mobile phase was filtered by using 0.2 μm filter. The flow rate of the mobile phase was maintained at 0.2 mL min^−1^ and water was used as sample diluent. The column temperature was 25°C and eluents were monitored at 210 nm. The injection volume for samples and standards was 0.5 μL. The total analysis run time was 10 min.

### Reagents

Bulk sample of SP and MH were received from the research development department of Dr. Reddy’s laboratories limited, Hyderabad, India. Commercially available Janumet tablets were manufactured by Merck & Co., Inc., NJ, USA. Hexane-1- sulfonic acid sodium salt and acetonitrile (HPLC grade) were purchased from Merck specialties private limited, India. Water was deionized and purified on a Milli-Q^®^ water purification system (Millipore, Bedford, MA, USA) and used to prepare all solutions.

### Preparation of Solutions

#### Standard Solutions

A standard solution containing 50 μg mL^−1^ of SP and 500 μg mL^−1^ of MH was prepared by dissolving an appropriate amount of SP and MH in diluent. An impurity blend solution of Metformin impurity-1 & 2 and Sitagliptin impurity with 100 μg mL^−1^ concentration was prepared in diluent.

#### Sample Preparation

To prepare the sample stock solution, tablets of Junumet™, each containing 50 mg of SP and 500 mg of MH, were accurately weighed and transferred into a clean and dry mortar, crushed to a fine powder. An appropriated amount was transferred into a 100mL volumetric flask, diluted to volume with diluent and sonicated for 10 min obtaining the final concentration of 50 μg mL^−1^ of SP and 500 μg mL^−1^ of the active pharmaceutical ingredient. The solution was filtered through 0.45 μm Millipore PVDF filter.

### Validation procedure

Method validation was performed as per ICH guidelines [18] for simultaneous determination of SP and MH in the formulations. The following validation characteristics were addressed: linearity, detection limit, quantification limit, precision, accuracy, robustness and specificity.

### System Suitability Criteria

The system suitability was assessed by five replicate analyses of the drugs at concentrations of 50 μg mL^−1^ of SP and 500 μg mL^−1^ of MH. The acceptance criteria was not more than 2.0% for the RSD for the peak areas and not more than 1.5 for tailing factor for the peaks of the both the drugs.

### Specificity – Forced Degradation Studies

Forced degradation studies were performed on SP and MH to prove the stability- indicating property of the method. The stress conditions employed for degradation study of SP and MH include light exposure [19], heat (105°C), acid hydrolysis (0.1 N HCl), base hydrolysis (0.1 N NaOH), water hydrolysis and oxidation (3% H_2_O_2_). For light studies, the monitoring period was 10 days whereas for heat, acid, base and water hydrolysis it was 48 h. Oxidation was carried out for 24 h. Peak purity of the principal peak in the chromatogram of stressed samples of SP and MH tablets was checked using PDA detector.

### Linearity of Response

Linearity solutions were prepared from stock solution at five concentration levels from 25 μg mL^−1^ to 75 μg mL^−1^ for SP and from 250 μg mL^−1^ to 750 μg mL^−1^ for MH. The slope, Y-intercept and correlation coefficient were calculated.

### Precision

#### Repeatability (intra-day)

The precision of the assay method was evaluated by carrying out six independent assays of SP and MH (50 μg mL^−1^ of SP and 500 μg mL^−1^ of MH) tablets against qualified reference standard. The percentage of RSD of six assay values was calculated.

#### Intermediate Precision (inter-day)

Different analysts from the same laboratory evaluated the intermediate precision of the method. This was performed by assaying the six samples of SP and MH tablets against qualified reference standard. The percentage of RSD of six assay values was calculated.

#### Accuracy

The accuracy of the method was evaluated in triplicate at three concentration levels, i.e. 50%, 100% and 150% of target test concentration (50 μg mL^−1^ of SP and 500 μg mL^−1^ of MH) in tablets. The percentages of recoveries were calculated.

#### Limit of Detection (LOD) and Limit of Quantification (LOQ)

The LOD and LOQ for SP and MH were estimated at a signal-to-noise ratio of 3:1 and 10:1, respectively, by injecting a series of dilute solutions with known concentration.

#### Robustness

The robustness of an analytical procedure is a measure of its capability to remain unaltered by small but deliberate variations in method parameters and provides an indication of its reliability during normal usage. To determine the robustness of the method, the experimental conditions were deliberately changed. The resolution of MH and its impurity-2 was evaluated along with %RSD for five injections and tailing factors for SP and MH. The mobile phase flow rate was 0.20 mL min^−1^; to study the effect of flow rate on resolution it was changed to 0.18 and 0.22 mL min^−1^. The effect of column temperature on resolution was studied at 20°C and 30 °C (instead of 25°C).The effect of pH of the mobile phase on resolution was also studied at 5.3 and 5.7 (instead of 5.5).

#### Solution stability and Mobile phase stability

The stability of SP and MH in solution was determined by leaving test solutions of the sample and reference standard in tightly capped volumetric flasks at room temperature for 48 h during which they were assayed at 12 h intervals. Stability of mobile phase was determined by analysis of freshly prepared sample solutions at 12 h intervals for 48 h and comparing the results with those obtained from freshly prepared reference standard solutions. The mobile phase was prepared at the beginning of the study period and not changed during the experiment. The % assay of the results was calculated for both the mobile phase and solution-stability experiments.

## Results and Discussion

### Method Development and optimization of stability-indicating assay method

The main objective of the chromatographic method is to achieve the separation of metformin impurities (Impurity-1 & Impurity-2) from Metformin, Sitagliptin impurity from Sitagliptin and also major degradation products formed under varied stress conditions. Sitagliptin (pKa = 7.7) and metformin (pKa = 12.4) are basic in nature.

**Fig. 1:**
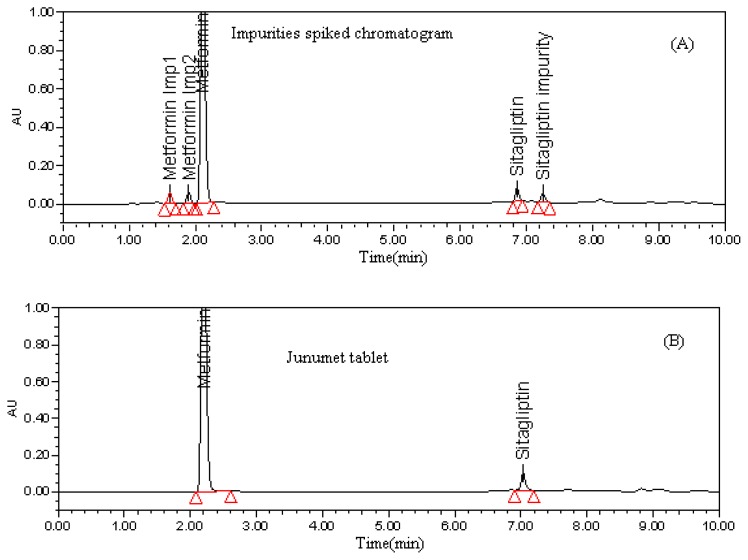
Typical chromatograms of (A) Impurities spiked chromatogram Tablet chromatogram (B) Tablet chromatogram. Chromatographic conditions: waters UPLC^TM^ BEH C8 (100 × 2.1 mm, 1.7 μm) column, mobile phase: buffer 10mM potassium dihydrogen phosphate and 2mm hexane-1-sulfonic acid (pH 5.5) and acetonitrile in gradient elution, flow: 0.2 mL min^−1^, column temperature: 25°C, injection volume: 0.5μl and detection: 210 nm.

Two different C18 (Aquity BEH C18, 100 × 2.1 mm; 1.7 microns and Zorbax C-18, 50 × 4.6 mm, 1.8 microns) Columns, one C8 Column (Aquity BEH C8, 100 × 2.1 mm; 1.7 microns) and Zorbax SB-CN, 50 × 4.6 mm, 1.8 microns columns were used for method development. For the initial trials Aquity BEH C18, 100 × 2.1 mm; 1.7 micron and Zorbax C-18, 50 × 4.6 mm, 1.8 microns columns were chosen with a mobile phase composition of phosphate buffer adjusted to pH 5.5 and Acetonitrile in various ratios in gradient mode. Good separation was observed between Metformin and Sitagliptin but no separation was observed between Metformin and its impurities and also the Metformin peak is early eluting. Further trials were carried out using Zorbax SB-CN, 100 × 4.6 mm, 1.8 microns and UPLC BEH C-8, 100 × 2.1 mm, 1.7 microns column using the same chromatographic conditions as above. In both cases, Metformin impurities were not separated from Metformin; only the retention time of Metformin was increased slightly on Cyno column. Different trials were carried out at different pH (2–7) of the mobile phase but all the attempts were futile. Since Metformin is a highly polar compound further trials were carried out using ion pair reagent in the mobile phase. Finally, good separation (USP resolution >2) between Metformin and its impurities along with good retention of Metformin (retention time around 2.2min) was observed using UPLC BEH C-8, 100 × 2.1 mm, 1.7 microns column with mobile phase consisting of a buffer (10mM potassium dihydrogen phosphate and 2mM hexane-1- sulfonic acid sodium salt, pH was adjusted to 5.50 with diluted phosphoric acid) and acetonitrile in a gradient program with a flow rate of 0.2 mL min^−1^. In the above mentioned conditions all the impurities related to Metformin and Sitagliptin are well separated along with good peak shapes (USP Tailing < 1.5) of Metformin and Sitagliptin. The retention times of MH and SP were found to be 2 min and 7 min, respectively. The blend chromatogram of Sitagliptin and Metformin with impurities (Metformin impurity-1, Metformin impurity-2 and Sitagliptin impurity) is shown in Fig. 1.

### Method Validation

Validation of an analytical procedure is the process by which it is established, by laboratory studies, that the performance characteristics of the procedure meet the requirements for the intended analytical applications.

### System Suitability

The system suitability test solution was injected and the chromatographic parameters like relative standard deviation for replicate injections of SP and MH and the tailing factor for both SP and MH peaks are evaluated. The relative standard deviation for replicate injections of both SP and MH was 0.30% and 0.40% for SP and MH, respectively. The tailing factor for both SP and MH peaks was 1.0 and 1.1 for SP and MH, respectively. This indicates the suitability of the system.

### Specificity – Forced Degradation Studies

**Fig. 2:**
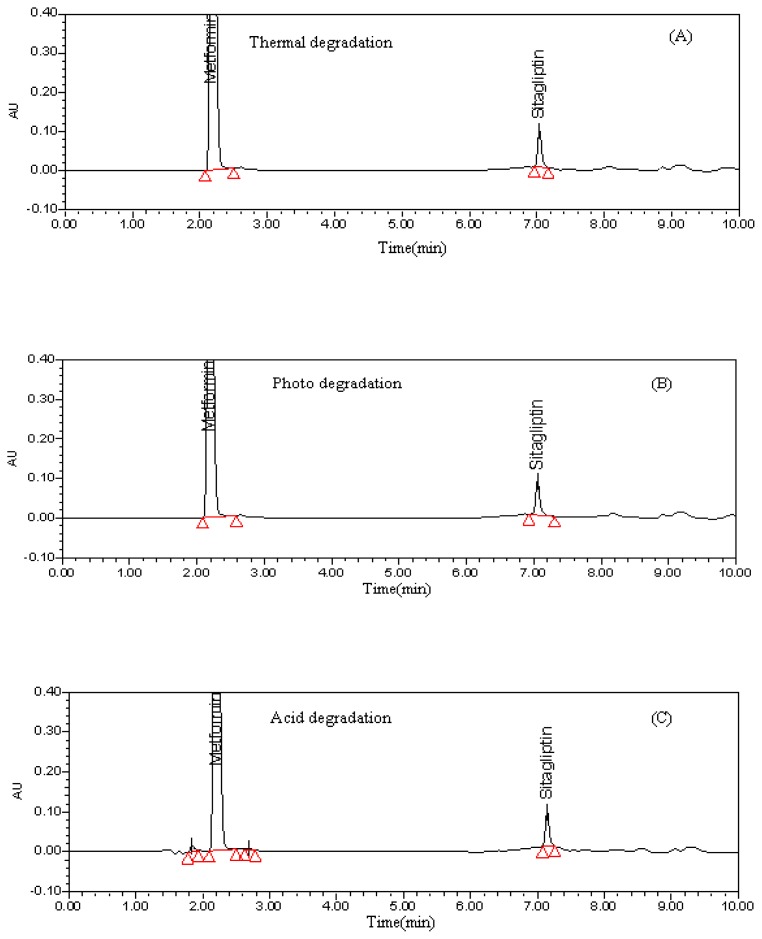

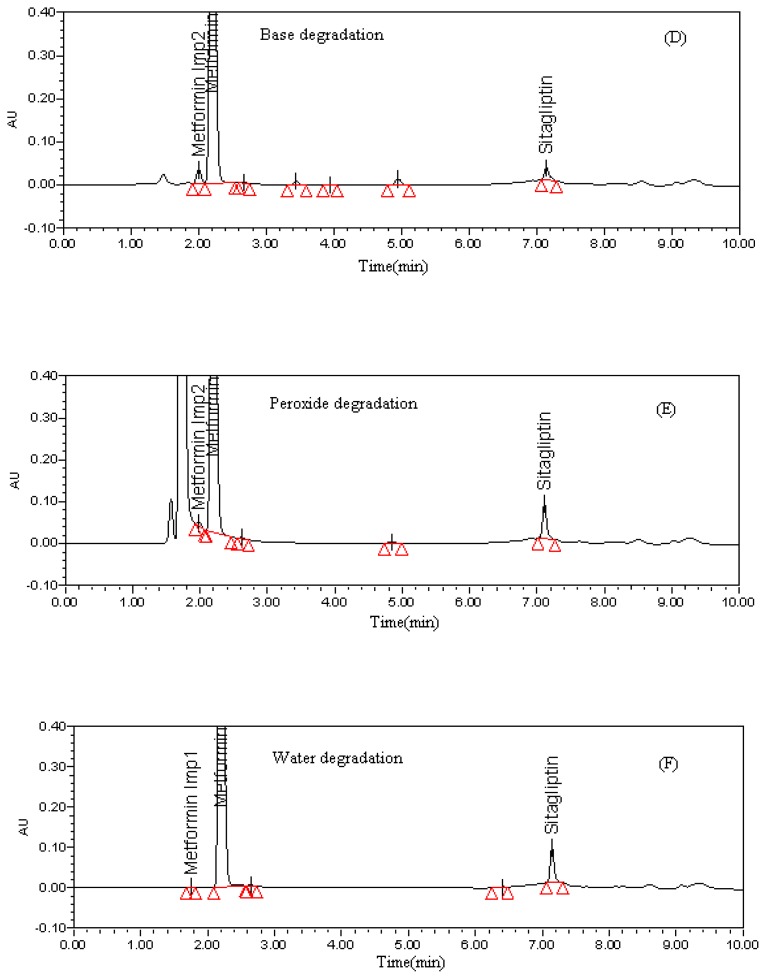
Degradation chromatograms (A) Thermal degradation (B) Photo degradation (C) Acid degradation chromatogram (D) Base degradation chromatogram (E) Oxidative degradation chromatogram (F) Water degradation chromatogram

Degradation was not observed in SP and MH stressed samples that were subjected to light and heat. However, the degradation was observed under base hydrolysis, acid hydrolysis, water and oxidative conditions. The peak purity test results derived from PDA confirmed that the SP and MH peaks were pure and homogeneous in all the analyzed stress conditions. This indicates that the method is specific and stability-indicating (Fig. 2). Purity angle for the selected drug components in all stress conditions was found to be less than the threshold angle. Data is recorded in Tab. 2.

### Linearity of Response

Linear calibration plot for this method was obtained over the calibration ranges tested, i.e. from 25 μg mL^−1^ to 75 μg mL^−1^ for SP and from 250 μg mL^−1^ to 750 μg mL^−1^ for MH, and the correlation coefficient obtained was greater than 0.999 for both drugs. The results show that an excellent correlation existed between the peak area and concentration of the analyte. The results are listed in the Table 3.

### Precision

The precision of an analytical method gives information on the random error. It expresses agreement between a series of measurements obtained from multiple sampling of the same homogeneous sample under prescribed conditions. The percentage RSD values for the precision study was 0.60%, 0.60% (inter-day precision) and 0.50%, 0.72% (intra-day precision) for SP and MH, respectively. This confirms good precision of the method.

### Accuracy

The percentage recovery of SP ranged from 99.75 to 101.27 and MH ranged from 98.32 to 100.60. Very good recoveries were made at each added concentration. Data is presented in Tab. 4.

### Limit of Detection (LOD) and Limit of Quantification (LOQ)

The limit of detection of SP and MH was 0.2 and 0.06 μg mL^−1^, respectively. The limit of quantification of SP and MH was 0.7 and 0.2 μg mL^−1^, respectively.

### Robustness

In all the deliberate varied chromatographic conditions (flow rate, column temperature and buffer pH), all analytes were adequately resolved and elution orders remained unchanged. The resolution between critical pairs, i.e. for MH and its impurity-2 was greater than 2.0 and tailing factor for SP and MH was less than 1.2. Data is presented in Tab. 5. The assay variability of SP and MH was within ±1.1%.

### Stability in Solution and in the Mobile Phase

Relative standard deviation (%) for assay of SP and MH during solution stability and mobile phase stability experiments was within 1.2%. The results from solution stability and mobile phase stability experiments confirmed that standard solutions and solutions in the mobile phase were stable for up to 48 h during assay determination.

### Tablet Application

Analysis was performed for commercially available innovator tablets. The mean assay (n = 6) for SP and MH was 100.20% and 99.62%, respectively. The percentage RSD value for the six assay values was 0.54%, 0.63% for SP and MH, respectively. The results are presented in Table 6.

## Conclusion

A simple specific stability-indicating UPLC method has been developed for the quantification of SP and MH simultaneously. This method has been validated and found to be specific, precise, accurate, linear, robust, rugged and linear for the detection and quantification of SP and MH. This method exhibited an excellent performance in terms of sensitivity and speed. The major advantage of this technique is that it is less time consuming and also eco-friendly because of its low consumption of organic solvents as compared to other analytical techniques. It helps in simultaneous estimation of SP and MH in pharmaceuticals i.e., in combination drugs. This method is suitable for routine analysis and quality control of pharmaceuticals.

## Figures and Tables

**Tab. 1:** Name, chemical structure and chemical name of SP, MH and 3 impurities (Sitagliptin impurity, Metformin impurity-1 and Metformin impurity-2)

No.	Name	Structure	IUPAC Name
1	Sitagliptin phosphate	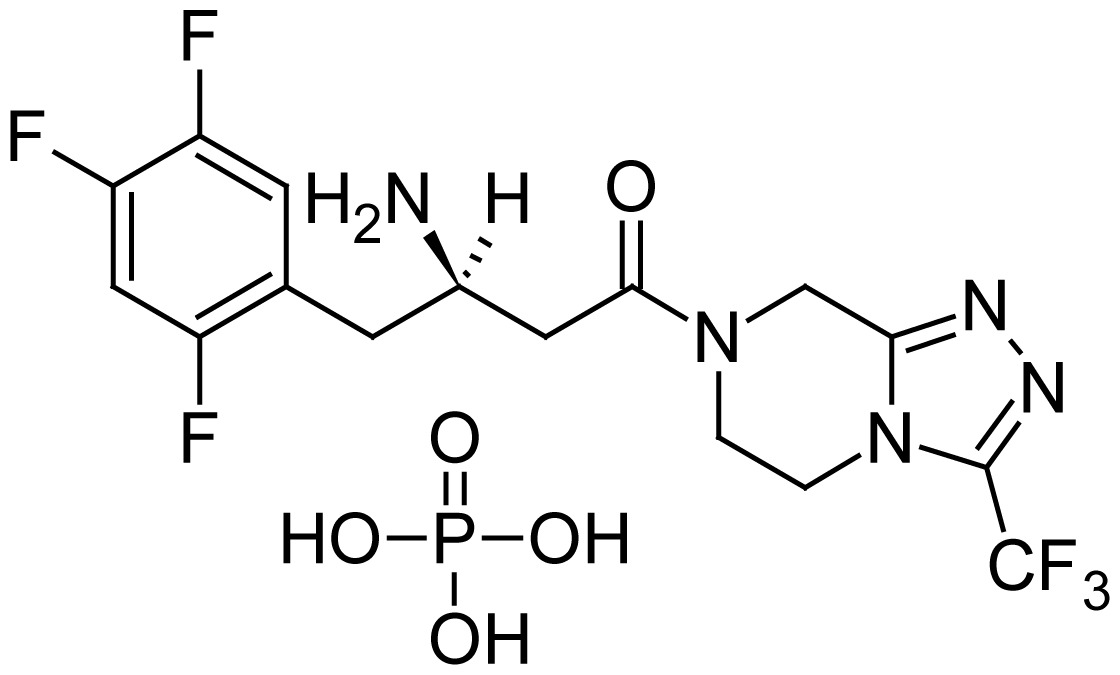	(3*R*)-3-Amino-1-[3-(trifluoro- methyl)-5,6-dihydro[1,2,4]- triazolo[4,3-*a*]pyrazin-7(8*H*)-yl]-4-(2,4,5-trifluorophenyl)butan-1-one phosphate
2	Metformin hydrochloride	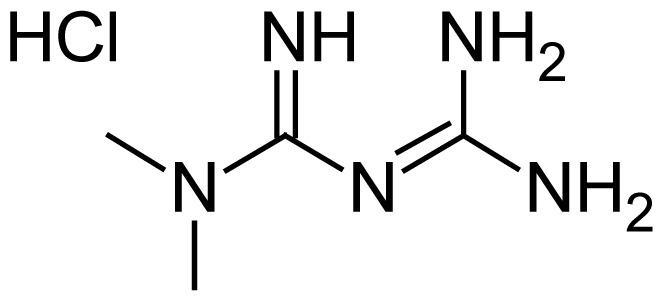	3-(Diaminomethylidene)-1,1- dimethylguanidine hydrochloride
3	Sitagliptin impurity	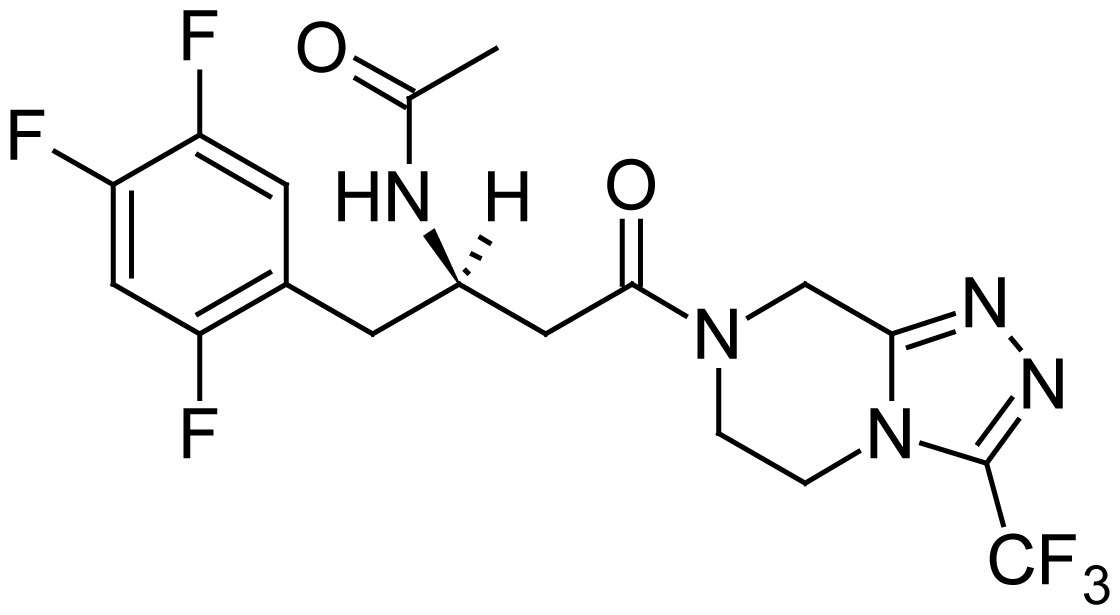	*N*-[(2*R*)-4-Oxo-4-[3-(trifluoro- methyl)-5,6-dihydro[1,2,4]-triazolo[4,3-*a*]pyrazin-7(8*H*)-yl]-1-(2,4,5-trifluorophenyl)butan-2-yl]acetamide
4	Metformin impurity-1	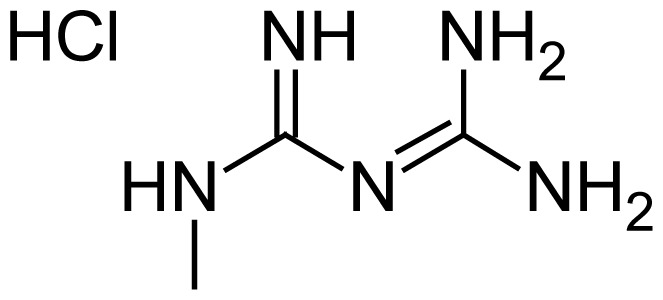	1-(Diaminomethylidene)-3- methylguanidine hydrochloride
5	Metformin impurity-2	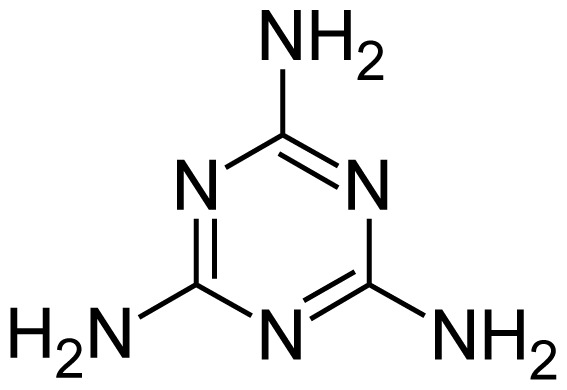	1,3,5-Triazine-2,4,6-triamine

**Tab. 2:** Degradation study data

Degradation conditions	% SP	Peak purity	% MH	Peak purity
Water reflux at 70°C for 48h	96.35	pass	95.50	pass
0.1 N NaOH at 70°C for 48h	35.06	pass	78.37	pass
0.1 N HCl at 70° for 48h	94.69	pass	94.01	pass
3% H2O2 for 24 h	87.80	pass	91.95	pass
Thermal (105°C for 10 days)	99.50	pass	99.58	pass
UV (254 nm for 10 days)	99.82	pass	99.27	pass

**Tab. 3:** Linear regression equations and correlation coefficient

Drug	Range (μg mL^−1^)	Slope	Intercept	Correlation coefficient
SP	250–750	3.975 × 103	1.7863 × 103	0.9999
MH	25–75	8.545 × 103	4.1987 × 104	0.9991

**Tab. 4:** Recovery of the assay method

Drug	Concentration (%)	% Mean recovery	% RSD
SP	50	99.75	0.45
SP	100	101.27	0.26
SP	150	100.18	0.29
MH	50	100.60	0.50
MH	100	100.06	0.47
MH	150	98.32	0.61

**Tab. 5:** System suitability parameters and robustness

Robustness parameter	Resolution between MH imp-2 and MH	Tailing factor		% RSD for 5 replicates	

		SP	MH	SP	MH
Buffer pH 5.30	2.4	1.1	1.1	0.41	0.50
Buffer pH 5.50	2.3	1.0	1.1	0.32	0.63
Flow rate 0.18 mL/min	2.2	1.0	1.1	0.33	0.42
Flow rate 0.22 mL/min	2.3	1.1	1.0	0.21	0.30
Column temperature 20°C	2.1	1.1	1.0	0.20	0.31
Column temperature 30°C	2.2	1.0	1.1	0.34	0.43

**Tab. 6:** Analysis data of tablet

Drug	Label claim (mg/tablet)	Amount found* (mg/tablet)	Assay (%)	% RSD
SP	50	50.1	100.20	0.54
MH	500	499.5	99.62	0.63

* Average of six estimations of tablet formulation.
